# Upward Trend in Dengue Incidence among Hospitalized Patients, United States

**DOI:** 10.3201/eid1705.101023

**Published:** 2011-05

**Authors:** Judy A. Streit, Ming Yang, Joseph E. Cavanaugh, Philip M. Polgreen

**Affiliations:** Author affiliation: University of Iowa, Iowa City, Iowa, USA

**Keywords:** dengue, viruses, trend, incidence, travel, hospitalizations, United States, dispatch

## Abstract

International travel and a global expansion of dengue fever have the potential to increase the incidence of dengue in the United States. We conducted a retrospective cohort analysis of trends in dengue among hospitalized patients by using the National Inpatient Sample (2000–2007); the number of cases more than tripled (p<0.0001).

The worldwide number of cases of dengue infection has increased nearly 35-fold in the past half-century, with a concomitant rapid geographic expansion (*1*). A large percentage of the world’s population is at risk for dengue fever: an estimated 2.5 billion persons live in virus-endemic areas. Each year, 50–100 million cases occur, hospitalizations for the infection have reached 500,000, and the global death toll is >20,000 persons (*2*). Large outbreaks have also occurred in close proximity to the US mainland (*3**,**4*). Despite the close proximity of these outbreaks to the United States, autochthonous cases in the continental United States have been relatively unusual, until the recent large autochthonous outbreak in Florida (*5**,**6*).

Risk for dengue infection to US residents has primarily been posed by travel. Among a multinational sample of ill travelers with a systemic febrile illness for whom a diagnosis could be determined, the GeoSentinel Surveillance Network reported that dengue fever was the second most common cause of such cases, behind malaria (*7*). A study from the same network reported that among travelers from all but 2 regions confirmed or probable dengue was more common than malaria (*8*). However, a recent published report indicated that, although reported cases of travel-associated dengue had increased during 1996–2005, “no significant trend” was shown (*9*). Another recent report showed a travel-associated increase, but this finding may have been due to, in part, the expansion of surveillance to include 2 independent monitoring systems (*10*). Because dengue has not been a reportable disease in the United States until recently, incidence and disease trends are difficult to determine. The goal of this study was to determine incidence of dengue fever among hospitalized patients and to analyze the recent trend in hospitalizations among patients with this disease.

## The Study

We conducted a retrospective cohort analysis of trends in dengue diagnoses among hospitalized patients using the National Inpatient Sample, the largest all-payer database of hospital discharges in the United States. The database is maintained as part of the Healthcare Cost and Utilization Project by the Agency for Healthcare Research and Quality and consists of a 20% stratified sample of discharges from nonfederal acute care hospitals (*11*). We first extracted all discharges from the National Inpatient Sample for hospital admission (i.e., the denominator of the incidence rate) from 2000 through 2007. Among this population, we then identified cases of dengue fever coded as either a primary or secondary diagnosis (i.e., code 061, dengue fever), according to the International Classification of Diseases, Ninth Revision, Clinical Modification.

For each yearly incidence rate, we calculated a 95% exact binomial confidence interval. To determine whether a significant trend in hospitalizations of patients with dengue fever occurred during the study period, we fit a logistic regression model using yearly incidence as the dependent variable and year as the independent variable. In addition, to accommodate the temporal association in the yearly incidence, we fit the model using generalized estimating equations, assuming an autoregressive correlation structure.

We also calculated the ratio of the yearly incidence rates at the beginning and the end of the study period (i.e., rates in 2000 and 2007). We tested whether this incidence ratio is significantly different from one using the Fisher exact test and computed a 95% exact confidence interval for the corresponding odds ratio using the hypergeometric distribution. (Because the incidence rates are fairly low, the odds ratio closely approximates the incidence ratio.)

Finally, we used the Monte Carlo variant of the Fisher exact test to investigate possible geographic variation in the incidence rate among the 4 US census regions. We tested for geographic homogeneity for every year in our 8-year sample. All statistical analyses were performed by using R version 2.10.1 (R Foundation for Statistical Computing; www.r-project.org) and SAS version 9.2 (SAS Institute Inc., Cary, SC, USA.).

During 2000–2007, ≈1,250 patients were hospitalized for dengue fever. The mean age of this population was 38 years (range newborn–87 years). The length of stay for these patients ranged from 0 to 35 days (median 3 days). For the Monte Carlo variant of the Fisher exact test, we found the incidence rates for the 4 US Census regions were homogenous for all years, except for 2004 and 2007. In these 2 years, the Northeast Region had the highest incidence rate (p<0.0001 for each year). Over the study period, the estimated number of dengue cases more than tripled from 81 cases in 2000 to 299 cases in 2007. The trend in the incidence of patients hospitalized with dengue during the study period was upward and significant (trend estimate 0.1313, model-based SE 0.0258; p<0.0001) (Figure). The increase from 2000 to 2007 was also significant (incidence ratio 3.5641, 95% confidence interval 2.0293–6.6232; p<0.0001).

**Figure:**
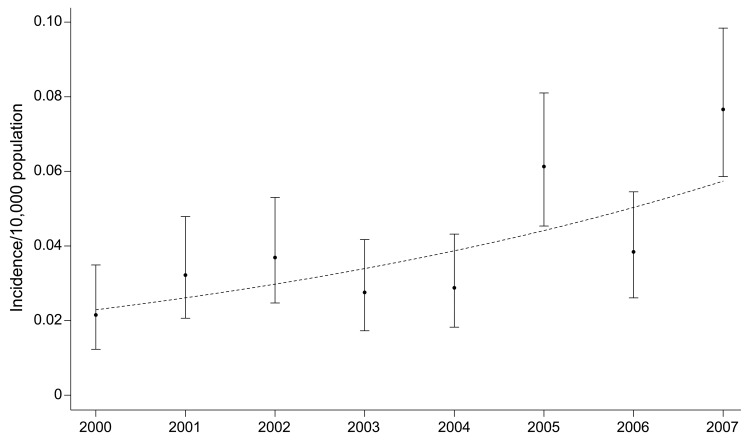
National estimates of dengue yearly incidence rates and 95% exact binomial confidence intervals (error bars), calculated by using data from the National Inpatient Sample, United States, 2000–2007. The trend (dotted line) is based on a logistic regression model fit by using generalized estimating equations. Note that the trend is curvilinear in the incidence rate, yet linear in the log odds of the incidence.

## Conclusions

We found a dramatic increase in the number of hospitalizations for patients with dengue fever in the United States. This increase is not surprising considering that 1) the number of cases in disease-endemic regions has increased in recent years, and 2) a substantial number of travelers annually enter the United States from the tropics and subtropics (*12*).

Although infrequent, severe consequences of dengue infection may occur in returning travelers. As individual travelers increasingly make multiple visits to dengue-endemic areas, the risk for severe dengue infections may similarly increase. A survey of 219 travelers who received treatment for dengue in Europe showed that 23 (11%) had severe clinical manifestations, including internal hemorrhage, plasma leakage, shock, and marked thrombocytopenia (*13*). We were unable to ascertain whether mosquito-borne hemorrhagic fever (International Classification of Diseases, Ninth Revision, code 065.4) also increased because the code appears quite infrequently, making statistical inferences unreliable. We also attempted to use deaths as a marker for disease severity, but we could not detect an increase in disease severity in our analysis because number of deaths was insufficient to accurately estimate a mortality rate.

Dengue and dengue hemorrhagic fever have been described as potential public health threats for residents of the US mainland (*14*). Despite the proximity of circulating dengue virus to the continental United States and the spread of the vector mosquitoes (*Aedes aegypti* and *Ae. albopictus*) to at least 26 states (*15*), autochthonous cases in the continental United States have been relatively rare (*5*) until the recent Florida outbreak. The increase in reported cases that we have documented highlights a potential risk for dengue spread within the United States. Although dengue fever was previously classified as reportable in some states, it did not become a reportable illness at the national level until 2010. Thus, some time is required before cases reported to public health departments can be used to establish reliable statistical estimates of national trends. Furthermore, the number of cases may not be linked to other relevant clinical data.

The major limitation to our study is that we used administrative data, and thus we did not have access to laboratory data or patients’ travel histories. In addition, milder cases treated on an outpatient basis were not captured. Nevertheless, our results indicate that the decision to make dengue fever a reportable disease in the United States was warranted and that increased vigilance focused on these new surveillance data is needed. In addition, administrative data, as we describe here, can be used to estimate the effects and severity of illness attributable to dengue.
